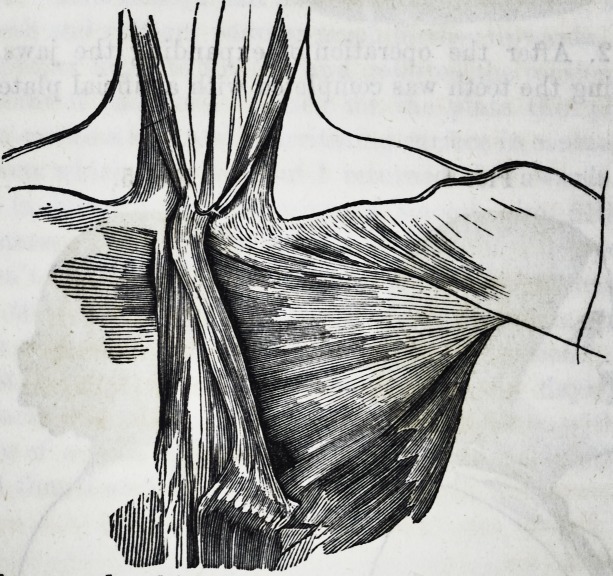# Extract from the Records of the Boston Society for Medical Improvement

**Published:** 1858-01

**Authors:** F. E. Oliver

**Affiliations:** Secretary.


### SELECTED ARTICLES.

### ARTICLE XIII.

*Extract from the Records of the Boston Society for Medical Improvement.* By F. E. Oliver, M. D., Secretary.

Oct. 12th.—*The Rectus Sternalis Muscle.*—Dr. Hodges showed the specimen, of which he read a description, together with a brief historical sketch of the muscle, from the pen of Dr. O. W. Holmes, from whom the specimen was received. The form and situation of the muscle may be seen in the accompanying cut, from a drawing by Dr. L. M. Sargent.

"The muscle which has been called *musculus thoracicus, rectus sternalis,* or *sternalis brutorum,* is well shown in this specimen from an adult white male. It arises, tendinous, from the anterior face of the upper bone of the sternum, passes downward and outward, tendinous and adherent at its inner edge; fleshy, and connected to the parts beneath by areolar tissue, in its outer portion; widens somewhat, and is inserted by tendinous slips into the cartilages of the third, fourth and fifth ribs. It lies over the inner edge of the pectoralis major and the intercostal muscles. The little force it can exert tends to raise the ribs and favor inspiration.

"Muscular anomalies, and especially supernumerary slips, are very often met with, and attract little attention. This muscle, however, is both physiologically and historically interesting.

"Physiologically, from its seeming correspondence with the thoracic continuation of the abdominal muscles seen in some of the lower animals, especially in the simiæ. But as that prolongation is placed beneath the pectoralis major, Theile considers the muscle we are examining as related to the sterno-mastoid. The axis of the muscle before us does not coincide with that of either, unless we should consider it as continued from the sterno-mastoid of the *opposite* side.

"Historically, this muscle is interesting from the attention which has been drawn to it by the extraordinary figure given by Vesalius, and the somewhat extensive literature belonging to it.

"In the fith plate of the second book of the great work '*De Corporis Humani Fabrica*' the recti muscles are represented as reaching to the first rib. In his first edition (*Basileæ*, 1543) Vesalius alludes to this formation as belonging to the simiæ. In the second edition (1555) he adds, 'this broad tendon and fleshy portion is the muscle which Galen calls the fifth of those moving the thorax, which is by no means so obvious in man as in caudate simiæ and in dogs. But we have delineated it for the sake of understanding Galen,' &c.

"Vesalius confesses, on the same page, that being accustomed to swear by Galen, he had favored his opinions more than he ought to have done, at the time when the drawings were made.

"Galen says, speaking of the recti, 'Their summit is a membranous tendon ascending to the upper part of the chest. This, however, naturally escaped the professors of anatomy, because the pectoral muscles lie over it.'

"The modern literature of this muscle would occupy many pages. Meckel gives a page to it, with various references. Theile also mentions it and has a brief reference to Vesalius. The first of these authors speaks of the muscle as being met with pretty frequently, the second as not being rare. I do not remember any thing like this out of many hundred subjects that I have examined.

"John Bell says, in his easy and rather careless way, 'Vesalius, Albinus and Sabatier, were thought to have found the recti abdominis extending up to the throat. But it is now found that Vesalius had only represented the muscles of a monkey or of a dog, which are very long upon the thorax of a human subject. Sabatier, after revising his notes, retracts what he had said: and Albinus also is supposed to have seen only a production of the mastoid muscle extending down the breast: for irregularities of this kind have been found.'

"Several of the more extensive modern anatomical works do not refer to this muscle, and it may perhaps be inferred that it is not so common as Meckel and Theile seem to have found it. It is one of those anomalies which could hardly fail to attract attention."

Dr. Gay remarked that he remembered a case in which this muscle was found fully developed on both sides, in a male subject of moderate muscular development, it being continuous *superiorly* with the tendon of the *sterno mastoid*, *inferiorly* with the *rectus abdominis*, and situated along the inner border of the pectoralis.

Dr. Jackson was not inclined to regard this muscle as a portion of the rectus abdominis; first from its being unconnected with the latter; secondly, from the difference in the direction of the fibres of the two muscles; and thirdly, from its having so tendinous an origin, while the rectus is rather fleshy than tendinous in its character.

Dr. Hodges expressed himself surprised that the existence of this muscle is not more familiar ; it being alluded to in nearly all the books of anatomy, and accurately described by Sharpey and Quain.

With regard to its connection with the rectus, he quoted the opinion of Meckel, who speaks of the latter as a polygastric muscle, "this formation leading by an insensible gradation to the formation of a special, external, abnormal, sternal muscle."

He thought the existence of this muscular anomaly no more remarkable than that of other muscular slips, often found, and which are usually assigned to contiguous muscles, or are considered as analogues of certain muscles existing in the lower animals. It would seem to require no greater stretch of the imagination to find in this slip an analogy of the long rectus of brutes, than to see in the platysma myoides, which is a muscle of mastication and expression, an analogue of the panniculus carnosus. The great peculiarity in this instance would seem to be its obliquity, to which, so far as he knew, authors had not alluded.

[The account of the anomalous thoracic muscle given above, is presented to the reader as it was written for the Boston Society for Medical Improvement and laid before the members at the meeting of October 12th. Since this account has been in the hands of the Editors of this Journal, by one of those singular coincidences which often attract the attention of scientific observers, the muscle in question has seen fit to make itself notoriously prominent.

In the *Virginia Medical Journal* for November, is an article entitled, "Respiratory Muscle. Observed by Powhatan Jordan, M. D., of Washington, D. C.," accompanied by a figure representing it, and designating it as "Jordan's Muscle." It is described as "a very beautiful triangularly shaped muscle, arising from fifth costal cartilage of the side, by a triangular fleshy belly, becoming tendinous about two inches from its origin, passing upward obliquely across the sternum, and inserting by a long, fine, bright, silvery tendon into the tendon of the sterno-mastoideus of the left side, or rather, in common with that muscle, into the left superior portion of the sternum."—It had no fellow. The same gentleman met with a pair of muscles of similar general character in a subsequent dissection. The numerous professors who held an inquest over this myological phenomenon, tumbled over the leaves of "Sharpey, Harrison, Horner, Wilson, Bichat, Cruveilhier," and other anatomical authors, without finding any mention of it. An editorial note in the same journal, however, mentions a brief reference to a sternal prolongation of the rectus, in Cruveilhier's Anatomy.

In the mean time, since my note was in the editor's hands, Mr. Nichols, a student of Prof. Jeffries Wyman, has met with a well marked case of this muscular anomaly, of which he promises to read a description at the meeting of the Boyston Medical Society.

I had contented myself with little more than references to some of the leading authorities, and feel unwilling to take up more time with this foolish little muscle, which is good for nothing in a practical point of view, except to teach professors and editors to be circumspect in proclaiming novelties, and to add a shelf to their libraries. Meckel's account, (to be found with the description of the pectoralis major,) is so complete, and his work is supposed to be so well known everywhere in the original, or in Jourdain's translation, or our own Dr. Doane's English version, that the idea of naming the venerable anomaly after any individual of the recent geological formations, is as if one should call the *equus caballus* Mr. Smith's animal, or the *bos taurus* Mr. Brown's quadruped. We are willing, however, to attribute the indiscreet naming of the muscle to the engraver, and not the observer or his scientific friends, for it is simply called "respiratory muscle" in the text.

Those who are not tired of the matter may look at the fifth and sixth plates of Vesalius's second book for another of his

Galenic muscles (X. *Tertius thoracem moventium*, fifth plate, and T. sixth plate.) These old figures are suggestive, but probably wholly imaginary.

The figure accompanying my paper owes something to the artist's imagination, as he had only half of the sternum to draw it from. This bone had been sawed and the muscles of the neck partially removed when the muscle was described. It should have been added in the description that its tendon was continuous inferiorly with the sheath of the rectus, and that a mere trace of a corresponding muscle existed on the other side in the shape of a few longitudinal fibres.] O. W. H.

*Boston Med. and Surg. Jour.*

## Figures and Tables

**Figure f1:**